# Occupational Therapy for People Living With Dementia in the Community: Workforce Development Opportunities

**DOI:** 10.1093/geroni/igaa057.2763

**Published:** 2020-12-16

**Authors:** Michael Lepore, Erin Long, Joan Weiss

**Affiliations:** 1 LiveWell Alliance, Southington, Connecticut, United States; 2 Administration for Community Living, Washington, District of Columbia, United States; 3 Health Resources and Services Administration (HRSA), Washington, District of Columbia, United States

## Abstract

Establishing a workforce capable of delivering evidence- and team-based HCBS for the growing population impacted by dementia is of growing public health importance. Occupational therapy (OT), in collaboration with other disciplines, offers promise for supporting people with dementia to live well at home. However, neither uptake of team-based programs for people with dementia nor availability of providers who work in teams to support people with dementia are well understood. We reviewed information from three federal programs to improve understanding of team-based workforce development needs and opportunities. Findings indicate that interprofessional evidence-based interventions for people with dementia are increasingly implemented but geographically limited and development of the OT workforce’s dementia capability is nascent in interprofessional/interdisciplinary training. OT is a key profession delivering evidence-based HCBS for people with dementia, but substantial opportunity exists for workforce development, including education, training, financing, recruitment, retention, care coordination, and translation and implementation of effective care.

